# Medicine, Pharmacy, and Dispensing

**Published:** 1899-10-07

**Authors:** 


### Medicine, Pharmacy, and Dispensing.

It is reasonable, and it is but in accordance with the high aims of our profession, that many of the readers of introductory addresses should turn for their subject either to questions of ethics or to problems of high scientific interest—texts on which to build up sermons on the nobility of medicine as a career, or on the high position of medicine as a science. Not only is it well that the young man entering on the study of medicine should be made to feel that he is not merely going to learn a trade by which he may be able to earn so many pounds a year, but it is good also for those who, buffetted as they may have been in the struggle for life, still retain a love for their old school, to come back, perhaps with sons just entering the same career, and hear again the fresh profession of the old faith; to find that honour, science, and philanthropy are still held to be the mainsprings of action in the life of a physician, and to see that notwithstanding the growth of the mercantile spirit, and the intrusion of the "practical" into the very arcana of every science, those who embrace medicine are still being brought up to act as if monetary reward were the last thing dreamed of. Yet we must live, and to do this we must take fees and, what is more, exact them: nay, worse, we must in many cases even make up physic. This is the commercial side of a noble profession, and many of us would be glad to sever all connection with it, and give over to the pharmacist everything related to dispensing.

Here is a link which turns our thoughts to druggists, and leads us to refer to the inaugural address given by Dr. Leech, of Manchester, at the Pharmaceutical Society's School of Pharmacy. He pointed out that, just as medicine, by a process of evolution, had parted with some old functions and had acquired new ones, so pharmacy, if only its educational advances were continued, might cast off some unprofitable functions, and become in the future increasingly pleasant, increasingly useful to the public, and increasingly profitable to those who practised it. We are sure that we wish to the druggists all the good things which Dr. Leech thinks likely to fall to their share, and, indeed, so far as wholesale manufacturers are concerned, we think that already their lot is cast in pleasant places. The outlook, however, for the pharmacist pure and simple—the druggist, that is, who would give up all "counter-prescribing" on the one hand, and all the rough trades that have grown up round his business on the other—is by no means so bright; and indeed we sometimes doubt whether retail pharmacy—the extempore preparation of separate bottles of medicine—is not an art of the past rather than one to depend upon in the future. We, quite as much as Dr. Leech, would like to see a general levelling-up which, while taking out of the hands of the pharmacist what Dr. Leech calls the "sale of articles unconnected with pharmacy," or, in other words, the dealing in wines, and oils, and paints, and general drysalteries, which so stamps him as the tradesman, would at the same time give to him the dispensing which at present is such a worry to the general practitioner.

It is an alluring prospect; but we are afraid it is hardly in accordance with the hard facts of life. As Dr. Leech points out, "the history of pharmacy shows that there has ever been a tendency among those engaged in it to take up the treatment of disease" and so far as we are able to judge, the better education of the modern pharmacist has by no means lessened the activity of this tendency. The proposal that medical men should, as a class, give up all dispensing to the chemists hinges largely upon this—Will they in turn give up dabbling in the treatment of disease? We doubt it. The very essence of the trading instinct is to take advantage of every "opening" that shows itself, success depends on energy and push, and we no more expect to see a chemist throw away the advantages which his education gives him in regard to counter practice, than we do to see him, as Dr. Leech suggests, give up "the sale of articles unconnected with pharmacy," so long as they afford a decent profit.

It is, we think, too much the habit of medical men who discuss this question to take for granted that, in giving up dispensing, they would be conferring a great favour, in return for which the pharmacists might be inclined to tie their hands in regard to counter prescribing. Once on a time this may have been so. But times are changed; the coated pill, the patent tablet, and the ready-made medicine are things that have now to be reckoned with, and they greatly alter the condition of the bargain. As the price of these things gradually comes down we hardly think that the chemist will greatly care for the honour of "dispensing" such medicaments, especially as in doing so he might have to compete with grocers and other tradesmen. There is no pharmacy in selling a score of tablets, or a bottle of So-and-so's cough drops. In fact, it seems not impossible that the steadily extending use of ready-made physic may tend to fasten still more firmly on the general practitioner the fetters of dispensing. On the one hand, it tends greatly to lessen the labour of the process, and on the other hand it obviously cuts away the only *quid pro quo* by which the chemist might perchance be tempted to give up dabbling in medicine.

